# Global climate change and human health: Pathways and possible solutions

**DOI:** 10.1016/j.eehl.2022.04.004

**Published:** 2022-05-07

**Authors:** Qi Zhao, Pei Yu, Rahini Mahendran, Wenzhong Huang, Yuan Gao, Zhengyu Yang, Tingting Ye, Bo Wen, Yao Wu, Shanshan Li, Yuming Guo

**Affiliations:** a Department of Epidemiology, School of Public Health, Cheeloo College of Medicine, Shandong University, Jinan, China; b Department of Epidemiology and Preventive Medicine, School of Public Health and Preventive Medicine, Monash University, Melbourne, 3004, Australia

**Keywords:** Climate change, Health, Pathways, Adaptation, Mitigation

## Abstract

Global warming has been changing the planet’s climate pattern, leading to increasing frequency, intensity and duration of extreme weather events and natural disasters. These climate-changing events affect various health outcomes adversely through complicated pathways. This paper reviews the main signs of climate change so far, e.g., suboptimal ambient temperature, sea-level rise and other conditions, and depicts the interactive pathways between different climate-changing events such as suboptimal temperature, wildfires, and floods with a broad range of health outcomes. Meanwhile, the modifying effect of socioeconomic, demographic and environmental factors on the pathways is summarised, such that the youth, elderly, females, poor and those living in coastal regions are particularly susceptible to climate change. Although Earth as a whole is expected to suffer from climate change, this review article discusses some potential benefits for certain regions, e.g., a more liveable environment and sufficient food supply. Finally, we summarise certain mitigation and adaptation strategies against climate change and how these strategies may benefit human health in other ways. This review article provides a comprehensive and concise introduction of the pathways between climate change and human health and possible solutions, which may map directions for future research.

## Highlights

• Climate change affects health via natural disasters and socioeconomic pathways.
• Benefits of climate change on health may exist in certain areas.
• Socioeconomic and environmental factors may modify climate change-health pathways.
• Certain mitigation and adaptation strategies are suggested against climate change.

## Graphical abstract

**Figure:**
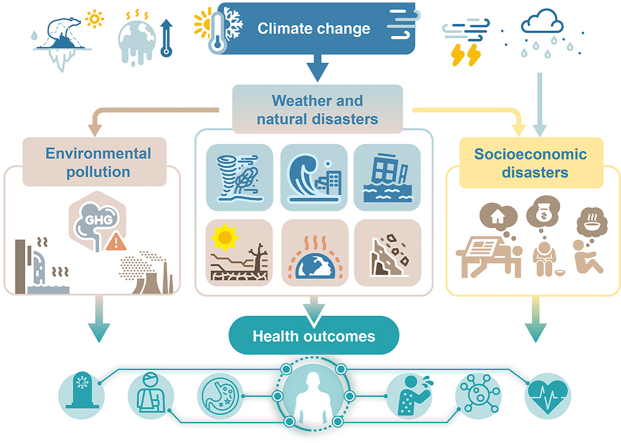

## 1 Introduction

The atmospheric concentrations of carbon dioxide (CO_2_), methane (CH_4_), and nitrous oxide (N_2_O) have increased by 47%, 156% and 23% since the beginning of the Industrial Revolution [1]. The last 30 years, paralleling the unprecedented emission of greenhouse gases, were recorded as the hottest period since 1850 [2]. The warming atmosphere and ocean over time have affected Earth’s climate system and disrupted the balance of nature through complicated pathways, resulting in climate perturbation and natural disasters, e.g., more frequent extreme temperature events (ETEs), droughts and floods, over the past decades [3,4].

Climate change has been regarded as the single largest global health challenge in the 21st century by affecting the physical environment and ecosystem and their interactions with human beings [3,5,6]. The health outcomes range from premature deaths caused by natural disasters to communicable diseases due to deteriorated hygiene and over-proliferation of pathogens [3,7]. A multi-country study has suggested that climate change is responsible for 400,000 additional deaths each year and will contribute to 700,000 annual deaths by 2030 [8]. All populations living in low to high altitudes and in low- to high-income countries are under the threat of climate change regardless of their age and socioeconomic status.

Although the health impacts of climate change have been well documented, so far, the evidence has primarily focused on health outcomes associated with certain single climatic conditions [9,10]. For example, previous studies have revealed that heatwaves could raise the mortality rate of both cardiovascular and respiratory diseases [11]. However, few studies have described the interactions between different climatic variables or with other non-environmental factors and their co-effects on human health. Clarifying the pathways is crucial for understanding the impacts of climate change on human health and developing mitigation and adaptation strategies.

In this article, we reviewed the major pathways between environmental hazards mediated by climate change and human health. We mainly focused on five sections: major signs of climate change; adverse effects of climate change; regional benefits of climate change; modifying effects of socioeconomic, demographic, and other factors; and certain mitigation and adaption strategies. For each section, we summarised the main findings and mapped out the potential directions for future research.

## 2 Major signs of climate change

The global mean surface temperature in 2020 has increased by 0.94 °C above the 1951–1980 average [12]. In the context of global warming, the frequency, intensity and duration of ETEs and natural disasters have been increasing substantially. For example, the total records of severe heatwaves between 2015 and 2020 have been twice as high as the 1951–1980 average [13]. In addition, 46% of floods, 34% of storms, 31% of droughts and 32% of wildfires occurred in the last 15 years since 1900 [13]. The mean sea surface temperature has risen at a pace of 0.06 °C per decade since 1880 [14]. The warming atmosphere has been melting glaciers and ice sheets, which reduces the heat reflection, and in turn, accelerates global warming. The melt of ice sheets and glaciers along with the thermal expansion of seawater plays a pivotal role in rising sea levels. Between 1971 and 2010, the sea level rose by 1.7 mm per year, and the rate has doubled since 1993 [15].

Greenhouse gases are currently emitted at a pace exceeding the worst projection of the Intergovernmental Panel on Climate Change (IPCC) [1]. Consequently, the mean surface temperature in 2100 is projected to be 4 °C higher than the 1986–2005 average, far beyond the previous estimation (2 °C). Forceful strategies, e.g., cutting off 70% of anthropogenic emission by 2050 and achieving net negative emission by 2100, may confine the warming trend under the representative concentration pathway (RCP) 2.6 scenario, but the temperature will still increase by 1.0 °C by the end of the 21st century [1]. Even if anthropogenic emissions can be stopped immediately, the global mean surface temperature will continue to rise by 0.2–0.5 °C in the following decade [16].

Accompanied by the global warming trend, the patterns of a variety of meteorological and other events are also projected to change in the future. For example, the global monsoon summer precipitation is expected to increase by 1.71% ± 2.38% under the high climate change scenario in 2021–2040 [17]. Changes in temperature and precipitation are associated with various flooding patterns, whose frequency by 2100 will be 4–14 times higher than the 1971–2000 average [18]. The modelling study also suggested that the drought intensity would show a widespread increase. Due to global warming, the frequency, intensity, and duration of ETEs are projected to increase [19]. Holding the present emission pattern of greenhouse gases, the sea level by 2100 will be 75 cm higher than the 1986–2005 average [20]. Whether climate change would increase the frequency of hurricanes remains unclear. However, climate change may exacerbate the hazard of hurricanes [21].

## 3 Main pathways between climate change and human health

**Fig. 1:**
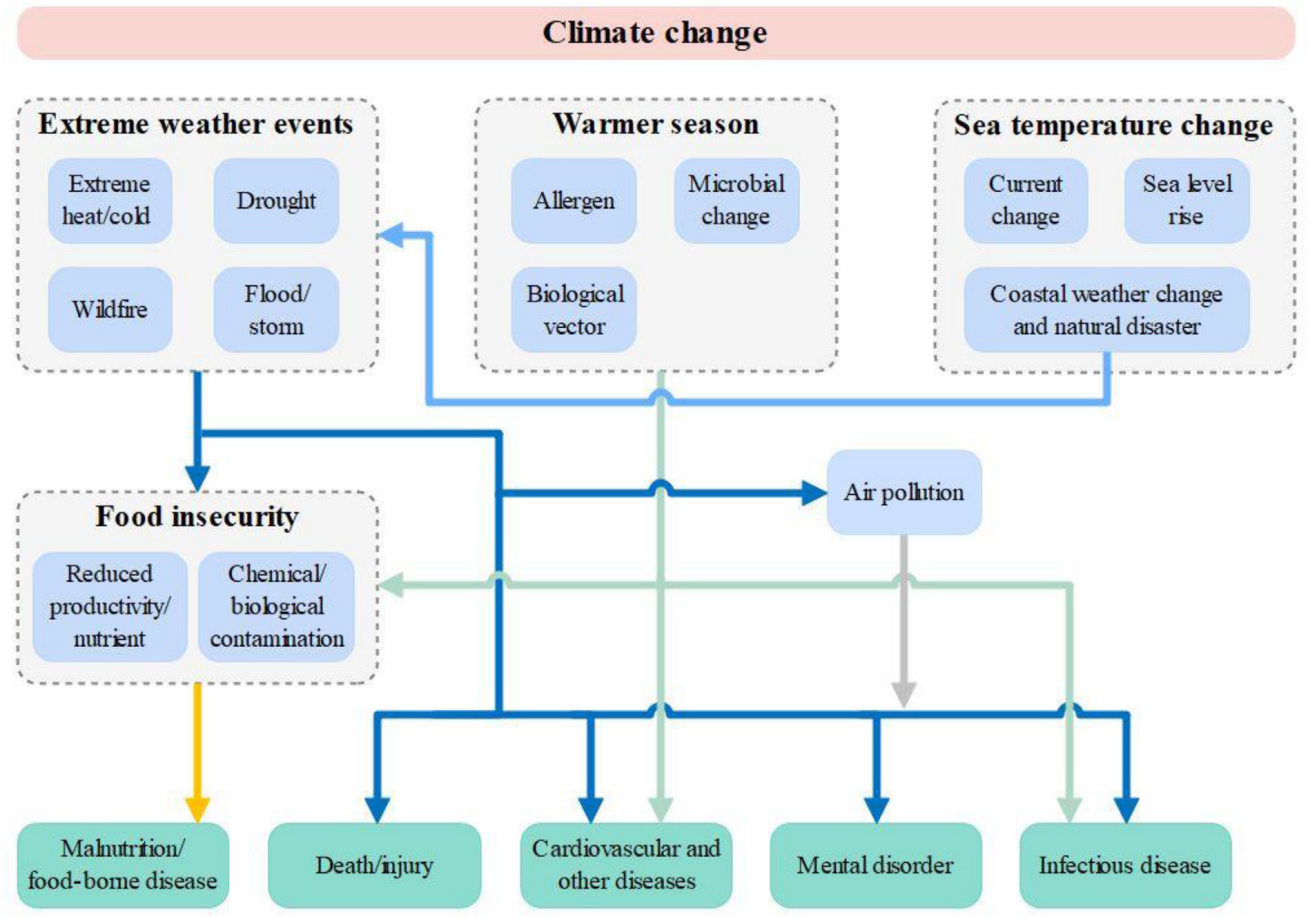
Main pathways between climate change and health outcomes.

Global warming causes anomalies in climatic and natural conditions, and their interaction may lead to a higher risk of various health outcomes (Fig. 1).

### 3.1 Extreme events

#### 3.1.1 Suboptimal temperatures

Both low and high temperatures are associated with increased mortality risk, exhibiting a J-, U- or V-shape [22]. It was estimated that 5,083,173 deaths per year were attributable to suboptimal temperatures, accounting for 9.43% of total deaths worldwide from 2000 to 2019. On top of that, the temperature-related excess mortality is expected to further increase until the 2050s [10,23]. In addition to excess deaths, the temperature change may cause a range of disorders. For example, exposure to extremely high temperatures has been associated with a higher possibility of emergency department visits and hospital admissions due to diseases of the cardiovascular, respiratory and metabolic systems [11,[24], [25], [26], [27]]. The increased cases of depression, anxiety, and suicide during hot days suggest that extreme high temperature is likely to trigger behavioural and mental disorders [28,29]. Evidence also showed that extreme cold temperature is strongly associated with cardiorespiratory outcomes and amplifies the hazards of hypothermia and traffic accidents [30,31]. Findings from some studies indicated higher susceptibility at the onset of hot or cold seasons when the human body has not yet acclimatised to extreme meteorological conditions [32]. Populations living in cold areas may be more susceptible to high temperatures than those in hot areas due to a lack of long-term adaptation and less awareness of the effects of extreme heat.

#### 3.1.2 Droughts

There have been 770 records of severe droughts since 1900, causing 38 million deaths [13]. Droughts are associated with extremely low precipitation, high evaporation and hot temperature, which may further increase the risk of dust storms, water insecurities, wildfires, food shortages and other health-related events. For example, dust storms may affect human health by carrying PMs, anthropogenic pollutants (e.g., dioxins, pesticides, and radioactive isotopes), and biomaterials [33]. The damage of dust storms may vary by geographic location. For example, researchers found that inhaling contaminated particles may contribute to valley fever in America, meningitis in West Africa, measles in Western China, and conjunctivitis in Asia [[34], [35], [36]].

Globally, four billion people experience severe water scarcity for at least one month each year [37]. Droughts may deteriorate water scarcity and water insecurity, increasing the risk of diarrhoea—the second leading cause of death, responsible for 525,000 global deaths each year in children under five [38]. In certain regions, every 10-mm decline in precipitation is associated with a 4% increase in diarrhoea incidence, while per 1 °C increase in temperature links to a 3%–11% increase in excess deaths from diarrhoea [39,40]. In 2050, there will be 320,000 deaths due to diarrhoea, of which 12% can be attributed to climate change, with 95% of cases occurring in Sub-Saharan Africa and South Asia [40]. Like other natural disasters, droughts may trigger conflicts and population migrations because of environmental deterioration and a shortage of essential resources [41,42].

#### 3.1.3 Wildfires

In addition to drought, high temperature and low precipitation can increase the risk of wildfire, extending fire season and burning time in some areas. Other climatic conditions, such as El Niño-Southern Oscillation (ENSO) are also associated with frequent droughts and wildfires in some coastal regions [43]. Direct health impacts of wildfire events include burns, injuries, mental health effects, and premature deaths [44]. Choking on gas and dust due to wildfire causes 339,000 additional deaths annually, far beyond the number directly caused by wildfires [45]. Wildfire smoke also contains toxic components, such as particulate matters (PMs) and nitrogen oxides (NO_x_) [46]. Studies have suggested a consistent association between the level of fire-induced PMs and the risks of death and hospitalisation from all causes, including cardiovascular and respiratory causes [47,48]. Some components, such as free radicals and benzene, are multi-organically toxic, leading to lesions and even malignancy of the digestive, hemopoietic, and reproductive systems [46]. Increased greenhouse gas emissions from burning and decreased biodiversity from forest loss are likely to worsen climate change [49].

#### 3.1.4 Floods and storms

Floods are usually caused by heavy rainfalls, storms and sea-level rise. Floods and storms are the most common and destructive natural disasters worldwide [50,51]. Between 1900 and 2015, there were more than 4500 records of floods, causing almost 90 million homelessness and 7 million deaths [52]. Besides injuries and drownings, threats continue after flooding. Studies have shown an increased short- and long-term risk of mortality and aggravation of non-communicable diseases in flood-stricken populations in the first year [53,54]. Pollutants deposited in the environment, such as river sediment and sewage, may be flushed out due to the flood. The overflow of sewers may contaminate drinking water and agricultural soil and water with chemicals, pharmaceutical ingredients and pathogens. Floods worsened the hygiene condition by destroying public facilities, increasing the risk of water- and vector-borne diseases. Heavy rainfalls and receding floodwater provided breeding sites for disease vectors, facilitating the transmission of malaria, dengue fever, yellow fever, and West Nile fever [55]. There were also reports regarding infections associated with ears, eyes, nose, skin, and respiratory and gastrointestinal tracts after flooding [56]. Moreover, the relationship between flood and mental disorders was also well documented, e.g., post-traumatic stress disorders (PTSD), depression, anxiety, and stress. Some studies estimated that the incidence of mental disorders might vary between 8.6% and 53% in the first two years after flooding [57]. Mental and physical disorders derived from floods may also affect maternal and offspring health, such as preterm birth, low birth weight, and impaired social functioning in their children [58,59].

Storms, such as hurricanes and tropical storms, could also significantly affect the environment and human health. It has been estimated that tropical cyclones have caused about 1.33 million deaths since the beginning of the 20th century, with more than 629 million people affected [60]. Similar to floods, in addition to the instant injuries directly caused by storms [61], studies have demonstrated an increased short-term risk of hospitalisations due to respiratory, infectious, and parasitic diseases and preterm birth [51,62]. Higher risks of mental disorders, such as acute stress, sleep disorder, and PTSD, have also been reported in storm-stricken populations by recent studies [59,63].

### 3.2 Ground-level air pollution

The effects of both short- and long-term air pollution exposures on cardiovascular mortality and morbidity have been ascertained according to previous controlled human exposure and epidemiological and animal toxicological studies [64]. The respiratory effects of air pollution exposure have been well investigated by large cohort studies, indicating that air pollution exposure causes decrements in lung function growth in children, lung function decline in adults, asthma, chronic obstructive pulmonary disease, lung cancer and respiratory death [[65], [66], [67], [68], [69], [70], [71], [72]]. Furthermore, accumulating evidence demonstrated that high exposure to air pollution could increase the mortality and the risk of endocrine diseases, metabolic diseases, nervous system diseases, reproductive diseases and cancer [69,[72], [73], [74]].

Climatic factors may be involved in the pathway from air pollution to human health [75,76]. High temperature promotes the chemical transformations of some air pollutants (e.g., the photochemical synthesis of ozone); air-stagnation episodes inhibit the dilution and dispersion of air pollutants, while rainfalls accelerate the deposition of air pollutants. On the other hand, climatic factors can influence an individual’s behaviour, which determines the way of exposure to air pollution. For example, in high latitude areas, a higher annual temperature may result in more days with an optimal temperature, on which outdoor activity will be encouraged, and individuals may be exposed to air pollution for a longer period. On the contrary, frequent rainfalls will result in a longer indoor time, which limits the exposure to outdoor air pollution but may amplify the effects of indoor air pollution. Some studies indicated that the adverse health effects of PM_10_, sulphur dioxide and nitrogen dioxide are more severe during cold days, while other studies had opposite findings [77]. It should be noted that air pollution can also significantly affect regional climate through aerosol direct effects (e.g., scattering or absorbing radiation) and aerosol indirect effects (e.g., participating in cloud formation), in addition to its role in global warming [78].

### 3.3 Temperature rise

#### 3.3.1 Vector-borne diseases

The survival and proliferation of pathogens and vectors may increase in a warming world, resulting in more epidemics of vector-borne diseases, mainly transmitted by arthropod vectors [79]. An extended warm season prolongs the epidemic period and enlarges the geographic distribution. Warmer temperature promotes oviposition and shortens egg incubation of vectors, and increases the risk behaviour of human beings, such as less dressing. Humidity is also associated with disease transmission by affecting the lifespan of vectors [80]. For example, female mosquitos may have a reduced size and wingspan in a warm environment with high humidity and require more frequent biting to survive [81]. Recent studies have projected that epidemics of vector-borne diseases are likely to rise in the following decades [82], in 2050, climate change will bring 33,000 additional deaths, with 97% occurring in East Sub-Saharan Africa and South Asia. It has been estimated that over 6.1 billion people will be at risk of mosquito-borne dengue fever in 2080, accounting for 60% of the world’s population [83].

#### 3.3.2 Allergens

The earlier onset of warm days in spring and delayed arrival of cold days extend the flowering period. In North America, elevated temperature and carbon dioxide concentration have resulted in a 20-day-earlier, 8-day-longer season of allergenic pollen, accompanied by a 21% increase in the level of annual pollen concentration between 1990 and 2018 [84]. A higher pollen concentration may be related to allergic rhinitis, asthma, hay fever and other allergic symptoms [85,86]. Oceanfront houses with rising sea levels and water-intruded houses after heavy rainfalls, storms and floods become damp and moist, increasing the growth of fungi and moulds indoors during warm days and thus leading to health problems such as respiratory allergies, asthma and rhinitis [87,88].

#### 3.3.3 El Niño, La Niña & Oceanic Oscillation pattern

Abnormal sea temperature and monsoon induced by global warming and other natural disasters may distract ocean currents and trigger certain events, such as El Niño, La Niña and Oceanic Oscillation patterns [89,90]. El Niño and La Niña contribute to the anomalies of temperature, precipitation and air pressure, which further cause natural or artificial disasters, e.g., droughts, floods, wildfires, storms and displacements. ENSO may also be associated with diarrhoeal diseases, especially cholera [91,92]. A previous study has also found that ENSO was likely to contribute to the outbreaks of dengue fever and malaria in some regions [93,94]. For example, a 1 °C change in sea surface temperature, i.e., a weak ENSO, was linked to a 20% increase in malaria in Colombia [94]. Similarly, La Niña has also been reported to be associated with an increased risk of epidemics such as malaria and infection of shigellosis and leptospirosis [91,95,96].

### 3.4 Food insecurity

#### 3.4.1 Crops

Climate change is projected to negatively affect food production and security in all aspects of the food system (availability, access, utilisation and stability) and their interactions. Extreme weather-related disasters, including droughts, heavy rainfalls, windstorms, floods and hails, are increasing, thus reducing the yields of major crops [97]. Besides, high moisture and temperature may proliferate pests and weeds, threatening food production and causing famine in some regions. Similarly, high indoor humidity results in food contamination via aflatoxin and other toxins of fungi and mould [98]. It has been estimated that 720–811 million individuals faced hunger in 2020 [99]. Consequently, human health is under threat due to the lack of sufficient nutrients to maintain life or being more susceptible to infectious diseases. By 2050, climate change will reduce 3.2% of global food availability per person, causing 529,000 additional deaths worldwide due to changes in dietary and weight-related factors [100]. Moreover, reduced food yield increases the use of fertilisers, herbicides and pesticides, further contaminating soil and water. Crops growing from contaminated soil and irrigation water can result in a broad range of food-borne diseases [101]. Last but not least, the global food system generates greenhouse gas emissions from multiple sources, with an average of 16 billion tonnes of CO_2_ equivalents per year from 2012 to 2017 [102].

#### 3.4.2 Fishery and aquaculture

Fish products provide 17% of animal protein intake and 7% of all proteins for the global population [103]. Climate change may affect the production ecology and biodiversity of aquatic systems, resulting in reduced production and yield, as well as changes in species composition in catches and geographic distribution. These changes are also affecting the socioeconomic status of the fisheries and aquaculture sector in many parts of the world [104]. Previous studies have estimated that the fishery yield may increase in some mid- and high-latitude regions but decrease in tropical areas because of global warming [105,106]. High sea surface temperature is a crucial environmental variable for the distribution, proliferation, toxicity and duration of harmful algal blooms (HABs) along certain coastlines [107]. The paralytic shellfish toxins (PSTs) excreted by HABs can cause deadly muscle paralysis and vomiting [108,109]. Besides, high sea surface temperature is likely to accelerate the proliferation of waterborne pathogens alongside coastlines, such as *Vibrio cholera*, *vulnificus* and *parahaemolyticus*. Consuming contaminated seafood can result in infections, including diarrhoea, septicaemia and even death. In addition to marine fishery, climate change may also shift the ecosystems of inland lakes and therefore affect the freshwater fishery. For example, a previous study investigated the long-term thermal change in 139 lakes across six continents, showing a 6.3% non-overlap between thermal habitats in baseline (1978–1995) and recent (1996–2013) periods [110]. Another study has estimated that 4%, 9% and 36% of freshwater fish species will have over half of their present-day geographic range exposed to climatic extremes in the future world with a 1.5 °C, 2 °C and 3.2 °C warmer global mean temperature, respectively [111].

## 4 Possible benefits of climate change on human health

Despite climate change being associated with a range of adverse health outcomes, certain climatic conditions may have some benefits [112]. For example, although rainfalls and floods may trigger vector-borne diseases in tropical and subtropical regions, heavy events may reduce disease outbreaks by destroying the habitats of vectors and their eggs. Similarly, some dry and low-latitude regions are projected to experience less precipitation due to climate change, which may reduce the density of insects, and hence, the prevalence of vector-borne diseases.

Climate change is also likely to reduce the prevalence of diseases and mortality associated with cold temperatures in certain countries and regions [113]. For example, with rising temperatures, labour productivity in countries with low baseline temperatures may increase, thus reducing cold-related labour loss [114]. In addition, certain countries may have less excess deaths associated with extreme cold ambient temperature in this warming world [113]. However, it remains inconsistent whether the decline in cold-related deaths can offset the increase in heat-related mortality in certain areas. Moreover, the positive effects of climate change vary substantially by geographical locations and tend to be short-term, which may be quickly outweighed by negative effects [115].

Warming temperature accelerates the melt of glaciers, thus providing more drinking and agricultural water for populations living in glacial-fed water catchments [112]. Longer warm days provide an extended period for plant growth, while a higher atmospheric concentration of CO_2_ increases photosynthesis by providing extra carbon sources. Over the last 35 years, 25%–50% of the vegetated land in the world has shown a greening trend largely due to the CO_2_ fertilisation effect [116]. The greening trend is more profound in high northern latitudes and northern temperate regions [117]. Increasing greening may also benefit human health as higher surrounding greenness is associated with a lower risk of many ageing-related diseases [118]. It should be noted that plants’ capacity to absorb nitrogen and minerals, such as zinc and iron, is expected to decline in some regions because of acid rain, leading to unbalanced food production, which may offset the benefit of climate change. Besides, extended precipitation may lead to delays in production processes [119]. For the marine fisheries and aquaculture sector, climate change may cause geographic shifts in fishery resources, with some regions in high latitudes experiencing an increase in productivity [120].

## 5 The impacts of socioeconomic, demographic and environmental factors on the pathways

The modifying effects of non-climatic factors on the climate change-health pathways should not be overlooked. Table 1 summarises some major modifiers, including age, sex, economic level, social status, education, land use, location, public health policy and public attitude.

**Table 1:** The main influence of major socioeconomic, demographic and environmental factors on the pathways between climate change and human health.

Aspects	Variables	Impacts
Demographic	Age	**Children and the elderly** (1) *Health status:* immature or impaired functions of the body’s physiological systems against climate change. (2) *Access to essential resources:* disadvantage in resource competition for medical care, food, freshwater and others.
	Sex	**Women** (1) *Access to essential resources:* disadvantage in resource competition and lower socioeconomic status. (2) *Exposure to climate-related risk factors:* higher exposure to indoor air pollution (e.g., black carbon) and less opportunity of escaping from floods and other natural disasters. (3) *Education:* poor education level and limited knowledge of self-protection from risks. (4) *Physiological differences*: hormones, organ size and build.
Socioeconomic	Socioeconomic and educational level	**The poverty** (1) *Access to essential resources:* less access to essential resources. (2) *Education:* limited knowledge of self-protection from risks. (3) *Response to climate change:* less awareness in mitigation and adaptation of climate change. (4) *Exposure to climate-related risk factors:* more outdoor work (e.g., farming, construction and mining) and higher exposure to outdoor air pollution and extreme temperatures.
	Public health policy	**Inactive policy** (1) *Access to essential resources:* insufficient resources for vulnerable populations. (2) *Health promotion:* poor health promotion regarding knowledge of self-protection from risks. (3) *Response to climate change:* passive attitude – less effectiveness of mitigation and adaptation. (4) *Technology*: less investment in clean energy and pollution-control technologies.
	Public attitude towards climate change	**Negative attitude** *Response to climate change:* passive attitude – less effective mitigation and adaptation.
Environmental	Land use	**Unbalanced ecosystem** (1) *Biodiversity loss:* more vector-borne diseases. (2) *Insufficient resources:* water and food insecurity.
	Topographic character	**Certain regions** (1) *City:* air pollution and temperature extremes (e.g., urban heat island). (2) *Coastline:* storm, sea-level rise, flood. (3) *Plain:* drought. (4) *Basin:* air pollution (e.g., trap pollution) and temperature extremes.

Specific populations are particularly vulnerable to climate change, partly due to socioeconomic inequalities, cultural norms, or intrinsic physiological factors. For example, aged populations and children are especially vulnerable to suboptimal temperatures due to impaired or immature thermoregulatory capacities [121]. New-borns and children have lower resistance to extreme disasters, pathogens, toxins and undernutrition and higher exposure per unit of weight to risk factors. Lower socioeconomic status often puts poor people at higher risk and reduces access to sufficient food and fresh water, high-quality medical services, and safe shelters against ETEs, floods and other natural disasters associated with climate change [112,122,123]. Health impacts of climate change also exhibit sex differences. Education levels, biological differences, social roles and activity patterns make women more vulnerable to environmental hazards [124]. The sex gap tends to be larger in countries where gender inequalities are particularly significant. Specific occupations may be particularly susceptible to climate change, such that farmers and construction workers have higher exposure to ETEs than others [125,126]. Certain places, such as cities, coastlines, plains and basins, may experience increased risks of heatwaves, floods, storms and droughts [127,128], which threaten human health through air pollution, infectious disease, food insecurity and undernutrition. Humans are experiencing an explosion in population size so that the global population by 2050 will be 29% larger than the 2011 level [129,130]. Agricultural production is one of the main sources of greenhouse gases. A larger population also means higher food demand and more anthropogenic activities, which irreversibly affect the climate, and eventually, show their joint impacts on human health. Therefore, non-climatic conditions can interact with each other and modify climate-health pathways.

## 6 Possible solutions

### 6.1 Mitigation

**Fig. 2:**
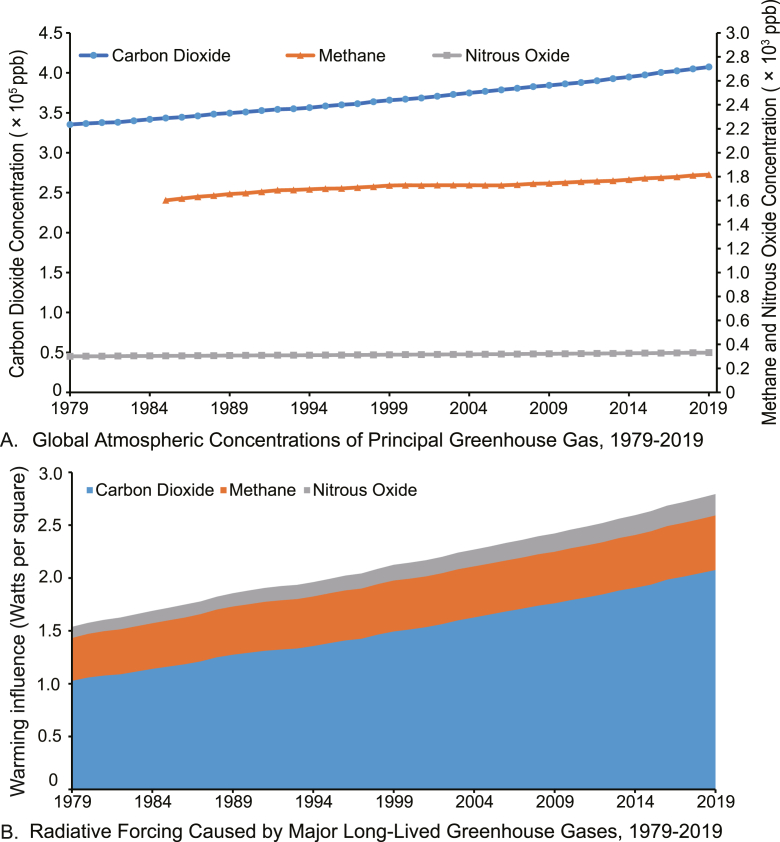
Global anthropogenic greenhouse gas emissions and radiative forcing in 1979–2019. Data are collected from the United States Environmental Protection Agency [133,134].

Global greenhouse gas emissions continued to grow to a record high of 52.4 gigatons of CO_2_ equivalent (GtCO_2_e; 59.1 GtCO_2_e, including land-use change) in 2019, with fossil CO_2_ reaching a record of 38.0 GtCO_2_e (72.5%), mainly originating from the main activity sectors (including power industry, combustion for industrial manufacturing and fuel production, transport, buildings and other sectors) [131]. Atmospheric concentrations of major greenhouse gases (CO_2_, CH_4_ and N_2_O) have increased dramatically over the last decades, with the emission of CO_2_ over 200 times and 1200 times higher than that of CH_4_ and N_2_O, respectively in 2019 (Fig. 2A). The degradation of CH_4_ and N_2_O may take decades, whereas CO_2_ exists in the atmosphere steadily [132]. The radiative forcing of the three gases was 2.8 in 2019, 48.5% higher than it was in 1990 (Fig. 2B). CO_2_ accounted for 74.3% of radiative forcing, with the rest shared by CH_4_ (18.5%) and N_2_O (7.2%).

Mitigation strategies are highly associated with atmospheric concentrations, lifetime and global warming influence of greenhouse gases, and their anthropogenic sources. Current estimates in Asia reported that air pollution reduction brought by mitigation actions under the 2 °C goal could decrease premature deaths by 0.79 million by 2050, with the mitigation co-benefits decidedly more than the cost [135].

#### 6.1.1 Reducing human emission

Although global CO_2_ emission has been projected to decrease in 2020 compared with 2019 emission levels because of COVID-19 [136,137], countries should strengthen their mitigation commitments in order to achieve the Paris Agreement’s long-term target of limiting global mean temperature increase to well below 2 °C [137]. Fossil fuel burning contributes the most to air pollution, which, in total, kills over 6 million people every year [9]. Globally, a phase-out of fossil fuel use can prevent an excess mortality rate of 3.61 million annually from outdoor air pollution [138]. Coal, oil and gas, the three most widely used fossil fuels, account for 85% of anthropogenic CO_2_ emissions. These fossil fuels are mainly consumed by electricity generation, heating, transportation and industrial production. From an energy supply aspect, transferring to clean and renewable energies such as solar, wind, geothermal and wave energy can effectively mitigate the emission burden. Strategies from an energy end-use aspect can contribute to the target of cutting down emissions, e.g., promoting and increasing investments in clean energy and adding health tax to energy prices. Transportation is responsible for 24% of the CO_2_ emissions from fuel combustion. To this end, effective actions should be taken, such as phasing out the internal combustion engine, reducing private car usage, encouraging walking, cycling and public transport, as well as creating people-centred cities with convenient public transport and high walkability to blue and green spaces. Since coal mining and gas and oil consumption produce one-third of atmospheric CH_4_, reducing the use of fossil fuels also mitigates the CH_4_ emission challenge. Given agriculture is another major source of CH_4_, changing cultivating strategy and food structure (e.g., improving livestock management, decreasing food waste and loss and adopting healthy and predominantly plant-based diets) can be more significant for cutting down CH_4_ emissions. Improving techniques for waste processing is another way to decrease CH_4_ concentration. Mitigation strategies of CH_4_ also effectively reduce N_2_O emissions because the two greenhouse gases share certain sources.

#### 6.1.2 Sinking or artificially removing atmospheric greenhouse gas

It is estimated that 61% of anthropogenic emissions of CO_2_ during 2010–2019 were removed via natural sink (35% by land and 26% by oceans, respectively) [139]. Forests and other green spaces are the main force for the land sink of CO_2_. Therefore, reforestation may reduce the atmospheric concentration of CO_2_ by increasing carbon sink, and so do algal blooms. Developing new technology to directly filter CO_2_ out from the atmosphere is also a practical way.

#### 6.1.3 Co-benefits of mitigation actions

The health co-benefits of mitigation actions can be obtained via various pathways. For example, facilitating cycling or walking is beneficial to human health by increasing physical activity and improving air quality via reducing fossil fuel combustion. Such efforts can achieve a reduction in adverse health outcomes (e.g., morbidity and mortality) related to air pollution (e.g., asthma), physical inactivity (e.g., obesity) and traffic noise (e.g., anxiety) [112,140,141]. Similarly, the promotion of residential greenness not only facilitates climate mitigation but also provides health co-benefits, such as increasing physical activities, improving mental health and reducing all-cause mortality [142]. Besides, energy prices, considering healthcare costs, may reduce the emission of greenhouse gases by over 20% and cut down deaths associated with outdoor air pollution by approximately 33% [143]. Given livestock farming consumes a large proportion of cereals, reducing meat consumption or increasing cultured meat may help ease food shortage. Last but not least, changing the current emission pathway to the RCP4.5 scenario will save around 500,000 lives per year by 2030 [52].

### 6.2 Adaptation

#### 6.2.1 General response

The adaptation strategies usually include four aspects: 1) to regularly assess health vulnerabilities and adaptation capacities, 2) to develop and implement an evidence-based adaptation plan for health, 3) to strengthen the climate resilience and environmental sustainability of healthcare systems and facilities,4) to protect health and advance climate justice by implementing health-promoting interventions in other sectors. More modules should be integrated into the early warning system, e.g., early forecasting extreme weather, predicting climate-related diseases, estimating possible health risks, evaluating damage levels, proposing preventative strategies, providing medical and rescue plans and establishing a program for post-disaster reconstruction [144].

#### 6.2.2 Specific actions

Specific actions can be taken at different levels to adapt to the impacts of climate change [145,146]. At the social level, public health systems and policies are critical, e.g., providing financial support and disaster-coping education for vulnerable populations. At the behavioural level, enhanced living conditions may reduce the impacts of extreme weather events. For example, a large number of deaths occurred in the elderly population during the 2003 European heatwave event, which could have been lessened if nursing homes and communities had been equipped with air conditioning [147]. At the physical level, increasing the coverage of green plants can promote the absorption of heat in cities. Greenspace may also reduce the morbidity of numerous disorders by blocking traffic noise, refreshing air quality, and providing sporting areas [148]. In addition, the urban areas are mainly made up of buildings, which contribute to the urban heat island. Some studies have provided certain primary evidence that urban adaptations, e.g., green roofs, cool walls and cool pavement, can effectively control urban climate, which may further cushion the adverse effect of extreme temperature events [149,150]. For example, a study in Sydney found that the peak indoor temperatures for buildings with or without green roofs and green walls were 27.7 °C and 34.6 °C, respectively [151]. Another study in China reported that the 2-m surface temperature in summer reduced up to 0.74 K and 1.19 K for green roofs and cool roofs, respectively [152]. Dams and reservoirs can protect people from floods and coastal storms, benefit commercial fishery and defend against droughts. Therefore, some physical-level adaptations not only reduce the impact of certain climatic conditions but also comprehensively enhance human health by maintaining a more comfortable environment and ecosystem.

## 7 Conclusions

To date, the signs of climate change are increasingly obvious, including ETEs, floods, storms and other climatic or natural disasters. These events affect human health from non-communicable diseases, infections, injuries to mental disorders, food insecurity and other issues through a variety of pathways. Certain socioeconomic, demographic and environmental factors may modify the pathways between climate change and health. Although climate change plays an adverse role at the global level, it can also benefit certain regions by improving local liveable ecosystems. For reducing the health threat due to climate change, mitigation and adaptation actions should be adopted at various levels, which may bring some co-benefits.

## Declaration of competing interests

The authors have declared no conflicts of interest.
